# Loop-mediated isothermal amplification assays for screening of bacterial integrons

**DOI:** 10.1186/0717-6287-47-53

**Published:** 2014-10-02

**Authors:** Guangchao Yu, Lei Chen, Chii-wann Lin, Bing Li, Hemiao Cui, Siyi Chen, Jian Miao, Huawei Bian, Dingqiang Chen, Yang Deng

**Affiliations:** First Affiliated Hospital of Jinan University, Guangzhou, 510620 China; College of Light Industry and Food Sciences, South China University of Technology, 381 Wushan Road, Guangzhou, 510640 China; Institute of Agro-products Processing, Anhui Academy of Agricultural Sciences, Hefei, 230031 China; Institute of Biomedical Engineering, National Taiwan University, Taipei, 10617 Taiwan; The Third Affiliated Hospital of Sun Yat-sen University, Guangzhou, 510630 China; Department of Laboratory Medicine, First Affiliated Hospital of Guangzhou Medical College, 600 Tianhe Road, Guangzhou, 510120 China

**Keywords:** Loop-mediated isothermal amplification (LAMP), Integron screening, Bacterial integrons, Class 1 integron, Class 2 integron, Class 3 integron

## Abstract

**Background:**

The occurrence and prevalence of integrons in clinical microorganisms and their role played in antimicrobial resistance have been well studied recently. As screening and detection of integrons are concerned, current diagnostic methodologies are restricted by significant drawbacks and novel methods are required for integrons detection.

**Results:**

In this study, three loop-mediated isothermal amplification (LAMP) assays targeting on class 1, 2 and 3 integrons were implemented and evaluated. Optimization of these detection assays were performed, including studing on the reaction temperature, volume, time, sensitivity and specificity (both primers and targets). Application of the established LAMP assays were further verified on a total of 1082 isolates (previously identified to be 397 integron-positive and 685 integron-negative strains). According to the results, the indispensability of each primer had been confirmed and the optimal reaction temperature, volume and time were found to be 65°C, 45 min and 25 μL, respectively. As application was concerned, 361, 28 and 8 isolates carrying *intI1*, *intI2* and *intI3* yielded positive amplicons, respectively. Other 685 integron-negative bacteria were negative for the integron-screening LAMP assays, totaling the detection rate and specificity to be 100%.

**Conclusions:**

The *intI1-*, *intI2-* and *intI3-*LAMP assays established in this study were demonstrated to be the valid and rapid detection methodologies for the screening of bacterial integrons.

## Background

In the past decades, indiscriminate abuse of existing antibiotics leads to proliferation of antibiotic resistance in microorganisms and consequently results in an increasing number of clinical failures in bacterial mediated diseases [1–3]. Up to date, a number of resistance mechanisms are responsible for the emergence and prevalence of antimicrobial resistance, such as plasmids and transposons [4].

Recently, the occurrence and prevalence of integrons in clinical microorganisms and their role played in antimicrobial resistance have been well studied. As a novel resistance determinant, integron was firstly reported in 1989 [5], and its mechanism and mobility, such as the excision and integration for gene cassettes, had been further investigated [5–14]. A complete integron platform may comprise three basic genetic elements, the integrase gene (*intI*), recombination site *attI* and a promoter (Pc). Pc is functionally demonstrated for all integrons and the integrase gene encodes a tyrosine-recombinase family integrase which mediates recombination between two recombination sites, mostly the proximal primary *attI* site and a secondary target called an *attC* site. Through specific excision and integration, gene cassettes become part of integron and mediate various function for the hosts, with resistance cassettes mostly identified [15–17]. Integrons have been classified and divided into several classes based on the differences and divergence in the sequences of *intI*. Up to date, 4 general classes of integrons have been identified and distinguished, and classes 1 to 3 integrons are known as multi-resistant integron (RIs). RIs had been reported to be capable of acquiring same gene cassettes via similar recombination platform [18–22]. Currently, integrons are considered to be widely distributed and spread among clinical microorganisms and thus play a key role in the dissemination of such antimicrobial resistance, which may eventually contribute to the unleashing of “Super Bugs” [23–25].

**Figure 1:**
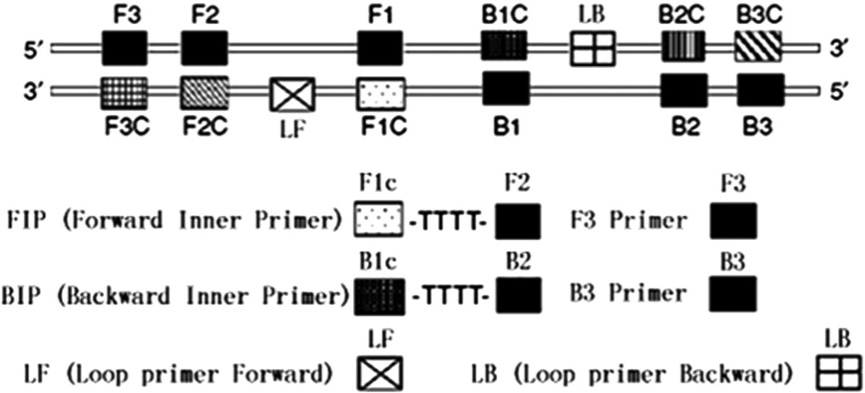
**Schematic diagram of primers used in the LAMP assays.** In detail, 6–8 distinct regions on every strand were used to design LAMP primers for the target gene. For the inner primers, the forward inner primer (FIP) consisted of the complementary sequence of F1 (F1c), a T-T-T-T linker and F2; the backward inner primer (BIP) consisted of the complementary sequence of B1 (B1c), a T-T-T-T linker and B2. The outer primers F3 and B3 located outside of the F2 and B2 regions, with loop primers LF and LB located beween F2 and F1 or B1 and B2, respectively. The scare bar is 10 nm.

As screening and detection of integrons were concerned, polymerase chain reaction (PCR) has been widely used. However, the requirement for PCR cycler machine and electrophoresis of PCR amplicons have restricted its further application, especially in clinical laboratory [2, 26]. In the latest decade, loop-mediated isothermal amplification (LAMP), as a novel nucleic acid amplification method, had been reported [27–29] and applied to the detection of various pathogenic organisms [26, 30–45]. This LAMP methodology relies on an auto-cycling strand displacement DNA synthesis performed by the *Bst* DNA polymerase large fragment, with 4 or 6 primers recognizing 6–8 distinct regions of the target gene (Figure 1) and generating the loop-mediated amplification under isothermal conditions between 60-65°C [27–29]. Amplicons are mixtures of many different sizes of stem-loop DNAs containing several inverted repeats of the target sequence and cauliflower-like structures with multiple loops [30, 46]. In this study, LAMP assays on resistance integrons screening (including class 1, 2 and 3 integrons) were evaluated, optimized and further applied to the detection of a large scale of clinical isolates, with approximately 60 min required for the entire process.

## Results and discussion

### Optimization of integron-screening LAMP assays

**Figure 2:**
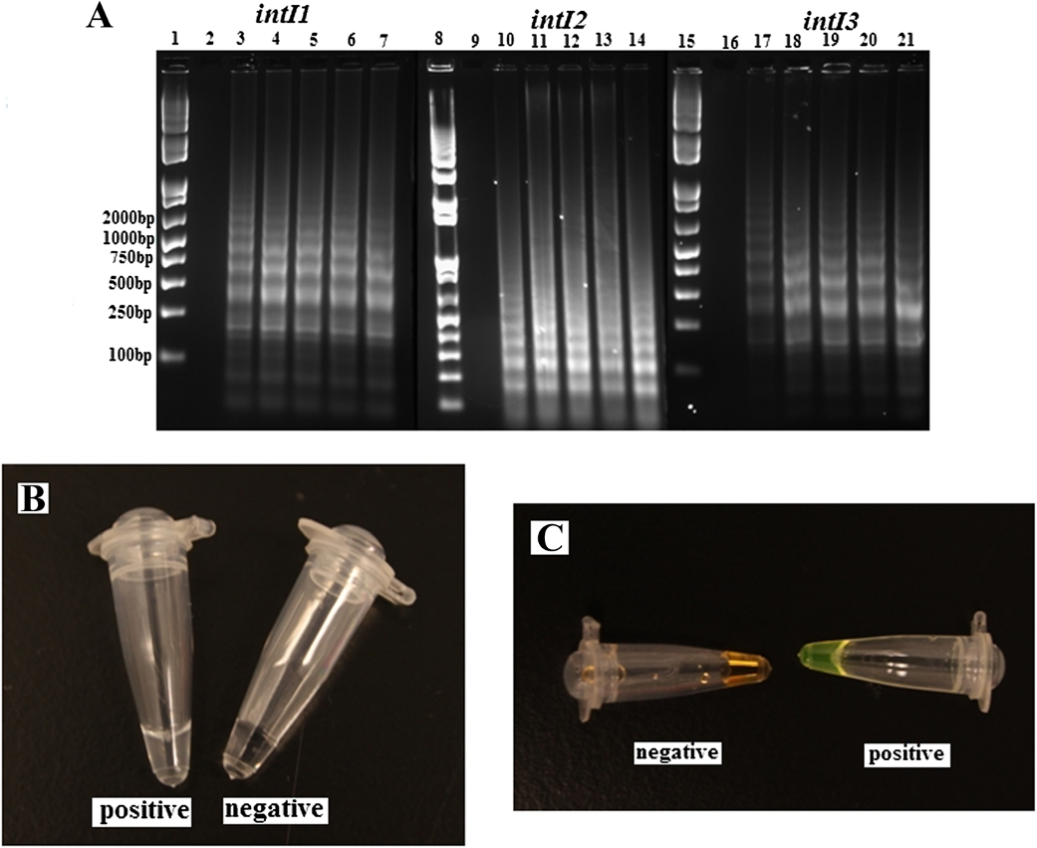
**Determination of LAMP amplicons by gel electrophoresis, as well as observation directly by naked eye and under UV light in combination with Sybr Green stain. A**: Monitoring of LAMP amplification by gel electrophoresis under different time points: lane 1–7, 8–14, 15–21 referring to LAMP assays of *intI1*, *intI2* and *intI3*, respectively. Lane 1, 8, 15: DNA Marker; lane 2, 9, 16: 15 min; lane 3, 10, 17: 30 min; lane 4, 11, 18: 45 min; lane 5, 12, 19: 60 min; lane 6, 13, 20: 75 min; lane 7, 14, 21: 90 min. **B**: LAMP products were visually detected by the turbidity derived from the white precipitate of magnesium pyrophosphate. **C**: LAMP products dyed with Sybr Green I were visually detected by examining color changes with the naked eye. Assays were performed at 65°C for 45 min.

Based on the amplification principle, the specific LAMP reaction generated many ladder-like pattern bands on agarose gel due to its characteristic secondary structure, with sizes ranging from 193 bp for *intI1*, 148 bp for *intI2* and 168 bp for *intI3*, respectively (Figure 2). LAMP assays were performed under isothermal condition between 59°C and 66°C and none of significant difference was found. Sequences of the small-size amplicons were identical to those PCR amplified with F3 and B3. However, the LAMP product amplified at 65°C exhibited slightly larger amount of DNA amplicons when compared to other temperatures (data not shown), which was consistent with previous studies [4, 26, 46]. Reaction lengths of LAMP assays were varied between 15 min, 30 min, 45 min, 60 min, 75 min and 90 min, under 65°C. With loop primers (LF and LB), the amplification was initially detected at 30 min, and reached maximal detection levels at 45 min. Nevertheless, without loop primers, amplification products were not detected until 90 min (data not shown). Primer specificity had been confirmed as no amplification was obtained in the absence of each of FIP, BIP, F3 or B3 primers, demonstrating LAMP assays were applicable only in the existence of both inner and outer primers. Therefore, each of the primers plays an indispensable role in auto-cycling strand displacement reaction by forming the loop out structure. All amplicons of integron-screening LAMP assays were determined by gel electrophoresis, as well as observation directly by naked eye and under UV light in combination with Sybr Green stain (Figure 2). The optimal reaction condition was determined as 65°C for 45 min, and then used for further LAMP assays.

### Sensitivities of integron-screening LAMP and PCR assays

**Figure 3:**
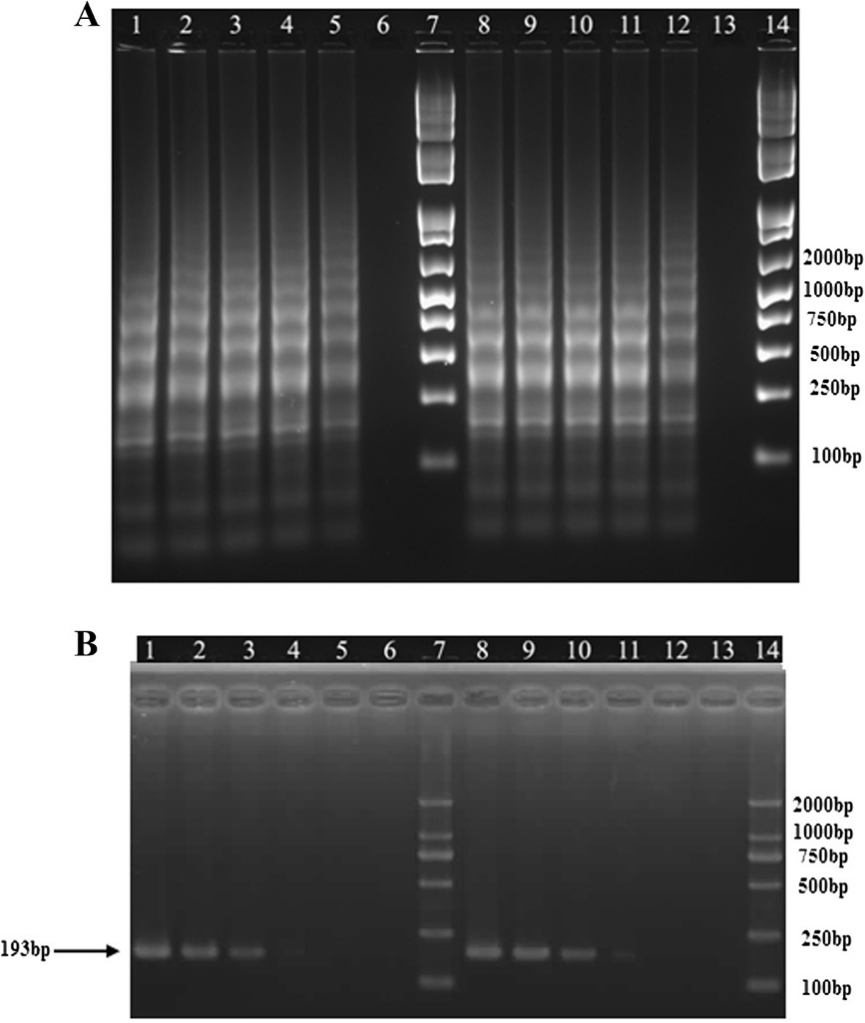
**Sensitivity of LAMP (A) and PCR (B) assays for detection of class 1 integron (** ***intI1*** **).** Lane: 1–6, 10^5^, 10^4^, 10^3^, 10^2^, 10, 1 CFU/reaction; 8–13: 1 ng, 100 pg, 10 pg, 1 pg, 100 fg, 10 fg DNA/tube; Lane 7 & 14: DNA Marker. LAMP assays were performed at 65°C for 45 min.

**Figure 4:**
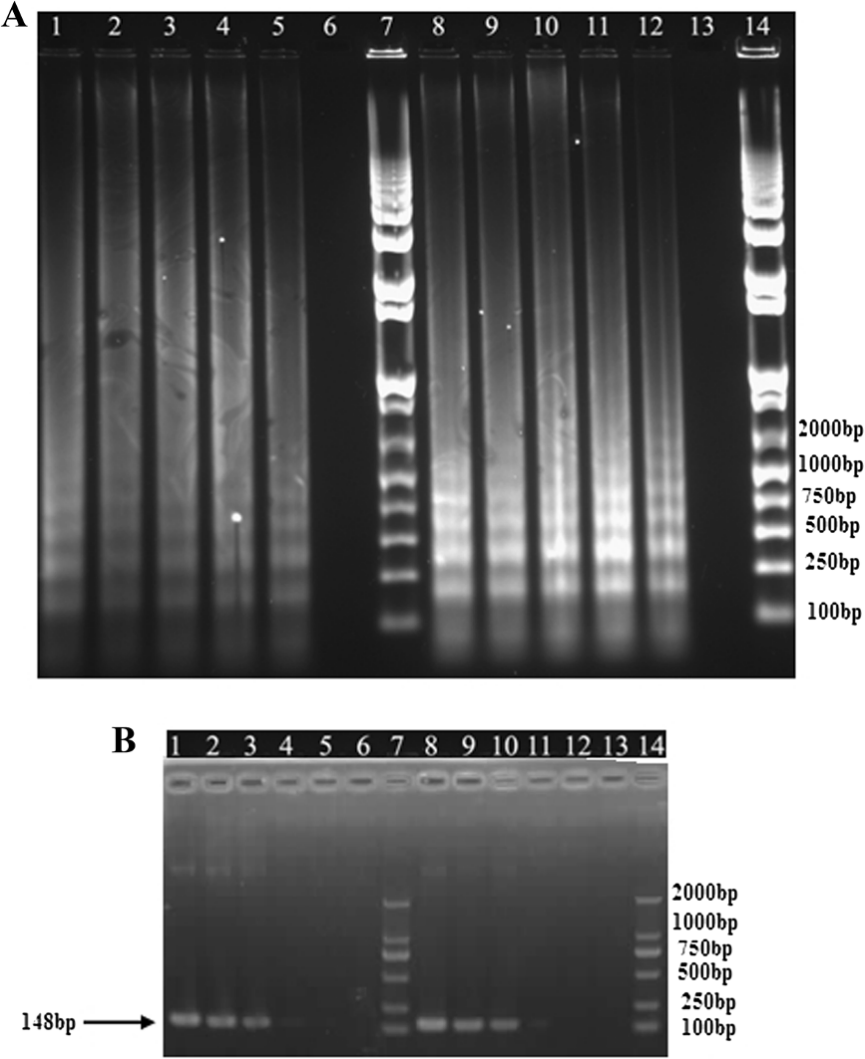
**Sensitivity of LAMP (A) and PCR (B) assays for detection of class 2 integron (** ***intI2*** **).** Lane: 1–6, 10^5^, 10^4^, 10^3^, 10^2^, 10, 1 CFU/reaction; 8–13: 1 ng, 100 pg, 10 pg, 1 pg, 100 fg, 10 fg DNA/tube; Lane 7 & 14: DNA Marker. These LAMP assays were performed at 65°C for 45 min.

**Figure 5:**
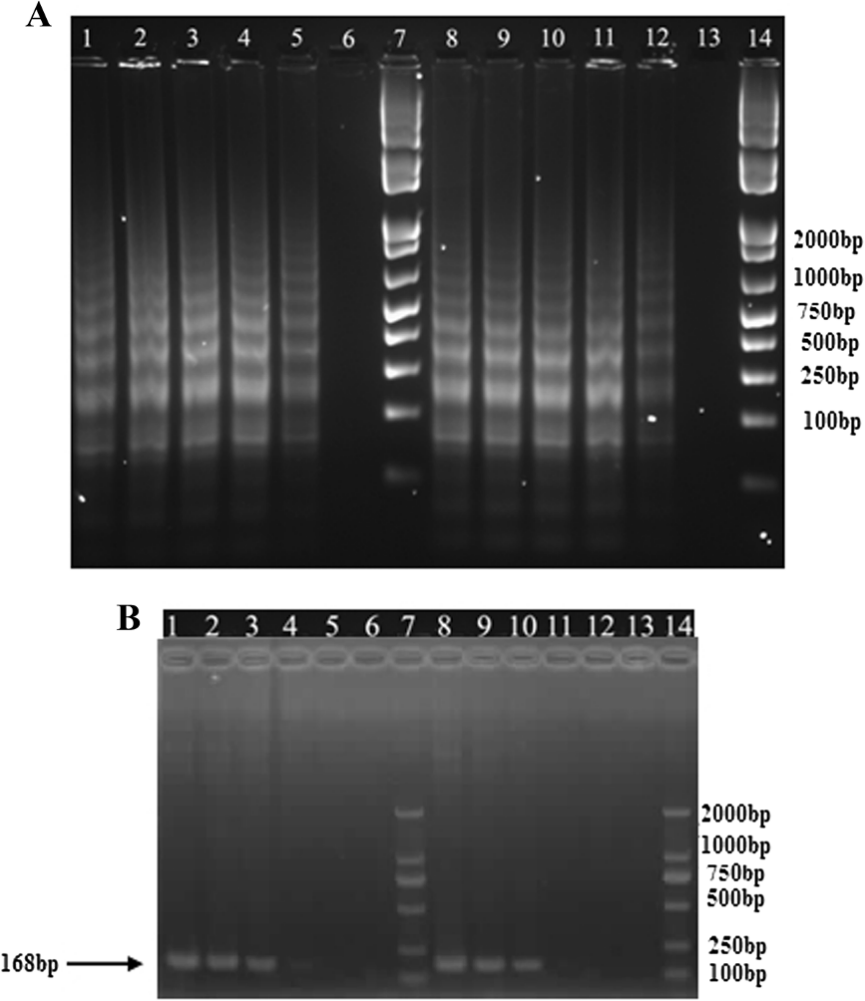
**Sensitivity of LAMP (A) and PCR (B) assays for detection of class 3 integron (** ***intI3*** **).** Lane: 1–6, 10^5^, 10^4^, 10^3^, 10^2^, 10, 1 CFU/reaction; 8–13: 1 ng, 100 pg, 10 pg, 1 pg, 100 fg, 10 fg DNA/tube; Lane 7 & 14: DNA Marker. These LAMP assays were performed at 65°C for 45 min.

The sensitivities of integron-screening LAMP and PCR assays were studied by the determination of both minimal CFU and minimal template DNA amount of bacteria. The detection limits of LAMP assays were found to be 100 fg DNA/tube and 10 CFU/reaction (LAMP was positive for sample containing 1 × 10^4^ CFU/mL, with 1 uL was included in the reaction system) for *intI1*, while PCR was 10 pg DNA/tube and 10^3^ CFU/reaction respectively, indicating that LAMP was 100-fold more sensitive than PCR assays (Figure 3). The same results were also obtained in the other two integron-screening LAMP assays for *intI2* and *intI3* (Figures 4 and 5). Due to its powerful amplification efficiency, LAMP had been characterized by high sensitivity and low detection limits, showing a significant advantage compared with PCR assays in the present study. The established LAMP assays may acceptably fulfill the requirement of low level detection of bacterial integrons in the clinical specimens.

### Application of LAMP assays on a large scale of bacterial isolates

**Figure 6:**
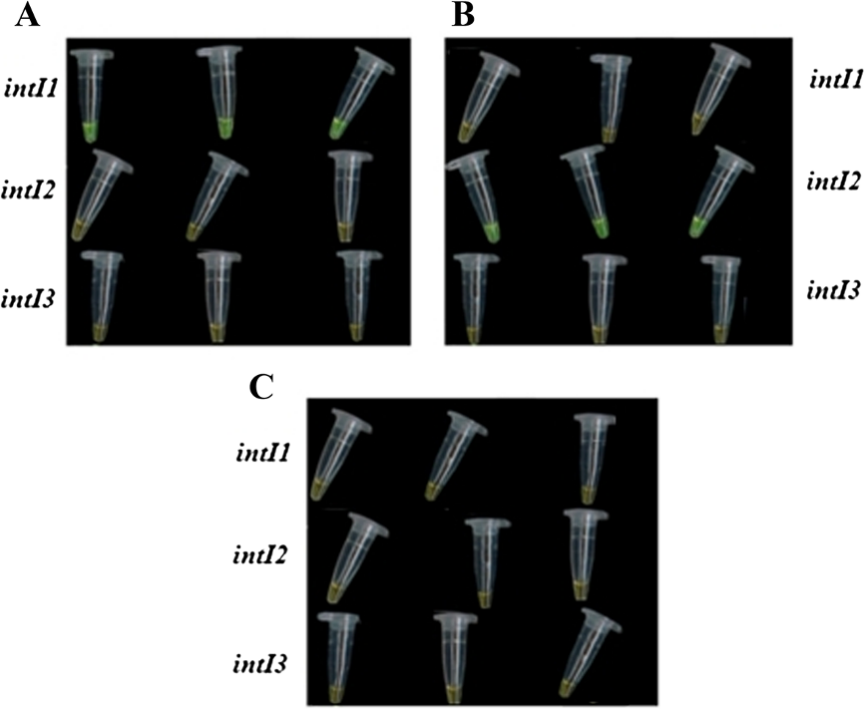
**Results determination through observation at the color change by naked eye when integron-screening LAMP assays were employed to detect** ***Staphylococcus warneri*** **012502 (A),** ***Escherichia coli*** **SK60 (B) and** ***Salmonella choleraesuis*** **ATCC 1312 (C) in triplicates.** Green indicates a positive result and orange indicates a negative result. LAMP assays were performed at 65°C for 45 min.

After establishment and optimization, such integron-screening LAMP assays were applied to the detection of 1082 microorganisms, including 397 integron-positive and 685 integron-negative isolates, with comparative validation by standard PCR assays (Table 1). For application, rapid DNA preparation process, simple heating equipments and results determination by observation directly by naked eye and under UV light had been applied. All of the 397 integron-positive isolates yielded positive amplicons and other 685 integron-negative bacteria were negative for the integron-screening LAMP and PCR assays, totaling 100% detection rate (Table 1). All the reactions had been replicated, and high reproducibility (100%) was obtained. For example, as shown in Figure 6, no difference was observed in the results of three parallel trials when the samples of *Staphylococcus warneri* 012502 (class 1 integron positive), *Escherichia coli* SK60 (class 2 integron positive) and *Salmonella choleraesuis* ATCC 1312 (three integrons negative) were detected in triplicates using integron-screening LAMP, respectively. Moreover, high specificity had been illustrated by no false positive observations for these reference strains during application in this study. With inner and outer primers recognizing six distinct regions, higher specificity should be achieved with LAMP as compared to more conventional PCR-based methodologies. Until recently, some other isothermal amplification techniques, such as nucleic acid sequence-based amplification and the self-sustained sequence reaction, were reported to be less specific due to their low reaction stringency (40°C) [26]. Furthermore, these technologies require either a precision instrument for amplification or an elaborate method for detection of the amplified products, which restrict their broad application [26].

**Table 1:** **Reference strains included in the evaluation of integron-screening LAMP assays**

Reference strains	No. of isolates	***IntI1*** ^a^	***IntI2***	***IntI3***
**Class 1 integron positive microorganisms**	361			
*Escherichia coli*	109	+	-	-
*Acinetobacter* spp.	21	+	-	-
*Pseudomonas aeruginosa*	51	+	-	-
*Klebsiella pneumoniae*	28	+	-	-
*Enterobacter cloacae*	13	+	-	-
*Staphylococcus aureus*	92	+	-	-
*S. epidermidis*	16	+	-	-
*S. haemolyticus*	5	+	-	-
*S. hominis*	9	+	-	-
*S. warneri*	1	+	-	-
*Enteroccus faecalis*	9	+	-	-
*E. faecium*	2	+	-	-
*Stretococcus* spp.	5	+	-	-
**Class 2 integron positive microorganisms**	28			
*Pseudomonas aeruginosa*	20	-	+	-
*Escherichia coli*	6	-	+	-
*Proteus* spp.	2	-	+	-
**Class 1 and 2 integrons positive microorganisms**	8			
*Pseudomonas aeruginosa*	3	+	+	-
*Enteroccus faecalis*	2	+	+	-
*Escherichia coli*	3	+	+	-
**Integrons negative microorganisms**	685			
*Staphylococcus aureus*	138	-	-	-
*S. epidermidis*	16	-	-	-
*S. haemolyticus*	7	-	-	-
*S. hominis*	8	-	-	-
*S. capitis*	1	-	-	-
*S. saprophiticus*	1	-	-	-
*S. sciuri*	1	-	-	-
*S. schleiferi*	1	-	-	-
*S. intermedius*	1	-	-	-
*Listeria monocytogenes*	58	-	-	-
*L. invanovii*	2	-	-	-
*L. welshimeri*	1	-	-	-
*L. seeligeri*	1	-	-	-
*Bacillus cereus*	6	-	-	-
*Escherichia coli*	121	-	-	-
*Vibrio parahaemolyticus*	108	-	-	-
*V. vulnificus*	1	-	-	-
*V. mimicus*	1	-	-	-
*Pseudomonas aeruginosa*	153	-	-	-
*Salmonella enterica*	44	-	-	-
*S. typhimurium*	2	-	-	-
*S. choleraesuis*	1	-	-	-
*S. enteritidis*	2	-	-	-
*S. typhi*	3	-	-	-
*S. paratyphi*	1	-	-	-
*S. aberdeen*	1	-	-	-
*S. gallinarum*	1	-	-	-
*Klebsiella pneumoniae*	1	-	-	-
*Enterobacter cloacae*	1	-	-	-
*Yersinia enterocolitica*	2	-	-	-
**Total**	1082			

^a^LAMP assays positive (+) and negative (−) results obtained in this study. Assays were performed in triplicates at 65°C for 45 min.

With the reaction performed under isothermal conditions without a thermal cycler, only simple equipment like a heat block and water baths were needed for the operation of LAMP assays at low expense. Additionally, the total detection time, including DNA preparation, LAMP reaction and results determination, was approximately 70 min, while conventional PCR methodology require nearly 2 h for amplification reactions alone. Therefore, the described LAMP methodology had been considered as the rapid, cost-effective, sensitive and specific detection assays for the screening of bacterial integrons.

## Conclusions

In this study, 3 integron-screening LAMP assays targeting on *intI1*, *intI2* and *intI3* were developed and further applied to the rapid detection of the bacterial integrons of 1082 clinical strains. Both sensitivity and specificity were found to be 100%. Comparing with conventional PCR, the *intI1*-, *intI2*- and *intI3*-LAMP assays exhibited advantages on detection limit, sensitivity, simplicity and rapidity. In conclusion, the described LAMP was demonstrated to be a valid and rapid detection method for integrons screening, which might aid in both the laboratory and clinical investigations.

## Methods

### Bacterial strains

For implementation and evaluation of the integron-screening LAMP assays, *Vibrio cholerae* O1 strain SK-10, *Escherichia coli* strain WF108314 harboring an R483::Tn7 plasmid and *Serratia marcescens* AK9373 were used as a positive control for class 1, 2 and 3 integrons, respectively. For application of the established LAMP assays, a total of 1082 strains were studied, including various species of gram-negative and gram-positive isolates (Table 1) with 397 integron-positive microorganisms and 685 integron-negative microorganisms, including 361, 28 and 8 isolates carrying *intI1*, *intI2* and *intI3*, respectively. All these tested bacteria were provided by the First Affiliated Hospital of Jinan University (Guangzhou, China) and Zhongshan Supervision Testing Institute of Quality & Metrology, and previously identified using standard procedures, including colony morphology, Gram staining, Vitek® 2 automated system and the API® commercial kit (BioMerieux, France) (unpublished data).

### Primer design

For each of *intI1*, *intI2* and *intI3*, a set of inner primers (forward and backward inner primers), outer primers (F3 and B3) and loop primers (LF and LB, to accelerate reaction) were specially designed for LAMP reaction to target 8 distinct regions (Figure 1 and Table 2). Forward inner primer (FIP)/backward inner primer (BIP) consisted of the complementary sequence of F1 (F1c)/B1 (B1c), a T-T-T-T linker and F2/B2; and outer primers F3 and B3 located outside of the F2 and B2 regions, respectively. Loop primers LF and LB were located between F2 and F1 or B1 and B2, which were designed to anneal at the loop structure of the amplicons and accelerate and enhance the sensitivity [27–29]. The primers were designed using PrimerExplorer® V4 (PrimerExplorer, Eiken Chemical Co. Ltd.) according to the reference sequences of *intI1*, *intI2* and *intI3* acquired on GenBank (Nos. AF550415, AP002527 and AY219651).

**Table 2:** **List of oligonucleotide primers used in this study**

Target	Sequence (5’ to 3’)	Size (bp)	Position	GenBank no.
***intI1***				AF550415
F3	AACAGTCTTGTACAAGTCCA	20	903-922	
B3	GGTGCTTTTGATATTTTTCCG	21	1075-1095	
FIP	CTCTCTTTCCTCTGCGGTCCTTTTGATGTTTTTCACACTTATTGGAT	43	936-958, 976-995	
BIP	TAAGGAATCACCTTGCAGATAAACTTTTTAGTACATTGGCATCGTGT	43	1000-1024, 1057-1074	
LF	CCAGAGTTAAGATTGAT	17	959-975	
LB	CGAAACAAGGCCAGTTTTTTACC	23	1028-1050	
***intI2***				AP002527
F3	TGTTGGAAGAATTTCTTTTGGA	22	726-747	
B3	GCTAATAGCCCTGCGTATC	19	924-942	
FIP	CGCGATGCATGATGATGACAATTTTTAGTGTTAATGCAATTCTGGGT	43	748-768, 788-809	
BIP	GAGCTTCCTTCTATGTGCCCGTTTTCAGAGTGGATGAGTCCCA	39	832-852, 895-912	
LF	TCGCACCGTAATTATGACT	19	769-787	
LB	AGATGGAAGAGTGCGTGGG	19	855-873	
***intI3***				AY219651
F3	TCGGTGTCTGTTATTAACCA	20	175-194	
B3	TGGAAACCGTTGTCACAC	18	371-388	
FIP	AGACGAAGATGGTCAAAACGCTTTTGCAGTTATTTTGCTGTGGA	40	206-224, 252-272	
BIP	CCGGGTTCGTTAATACGGCATTTTCGGGCACTGATATATGTGT	39	302-321, 352-370	
LF	TGATAGACATCAAGCCCTCGT	21	228-248	
LB	CAAATACTTTCTACCGTTTT	20	323-342	

### Preparation of template DNA

Cultural conditions and template DNA extraction of the tested gram-positive and gram-negative strains were prepared as described previously [47–52]. In brief, these strains were innoculated Luria-Bertani (LB) broth and incubated overnight at 37°C with shaking. The collected culture was then diluted 10-fold in 10 mM Tris–HCl (pH 8.0) containing 1 mM EDTA. The suspension was boiled for 10 min and further kept on ice. After centrifugation at 12,000 *g* for 3 min, the resulting supernatant was used as templates for LAMP and PCR assays.

### Implementation, evaluation and optimization of integron-screening LAMP assays

To implement and evaluate the integron-screening LAMP assays, *V. cholerae* O1 strain SK-10, *E. coli* strain WF108314 harboring an R483::Tn7 plasmid and *S. marcescens* AK9373 were employed as reference strains. Evaluation and optimization of this integron-screening LAMP included the investigation on mixture volume (3 volumes, with 12.5 μL, 25 μL and 50 μL), reaction temperature (8 temperatures, with 59°C, 60°C, 61°C, 62°C, 63°C, 64°C, 65°C and 66°C), reaction time (8 time points, with 15 min, 30 min, 45 min, 60 min, 75 min and 90 min), sensitivity (namely detection limit) and specificity of both the primers and integron types. LAMP assays were carried out in 3 different reaction mixture volumes, containing 1.6 μM (each) of the primers FIP and BIP, 0.2 μM (each) of the primers F3 and B3, 0.8 μM (each) of primers LF and LB (for each individual primer), 6 mM MgSO_4_, 1.6 mM of deoxynucleoside triphosphates, 1 × thermopol buffer (New England Biolabs, Ipswich, MA, USA), 1 M betain (Sigma, St. Louis, MO, USA) and different amounts of template DNA. For LAMP reaction, initiation was started by heating at 95°C for 3 min, followed by chilling on ice for 30 s with 1 μL (8 U) of *Bst* DNA polymerase (New England Biolabs, Ipswich, MA, USA) further added. After incubation at various temperatures ranging from 59°C to 66°C for 15 min-90 min, the reaction was terminated by heating at 80°C for 2 min. Simultaneously, PCR reactions were performed in parallel using the two outer primers F3 and B3, with the thermal profile as follows: 94°C for 5 min, 30 cycles of 94°C for 30 s, 50°C for 30 s, and 72°C for 30 s and a final extension cycle at 72°C for 7 min. The primers used in this study are listed in Table 2. The amplified products (5 μL/well) were analyzed by gel electrophoresis in 2% agarose gels and stained with ethidium bromide for 10 min. The detection limits of LAMP and PCR assays were ascertained by the determination of both minimal colony-forming units (CFU) and minimal template DNA amount of bacteria. In brief, overnight cultures and template DNAs from *V. cholerae* O1 strain SK-10, *E. coli* strain WF108314 and *S. marcescens* AK9373 were serially diluted 10-fold with sterile water, ranging from 10^2^ to 10^8^ CFU/mL and 10^−14^ to 10^−7^ g DNA, respectively. A negative control was performed using sterile water instead of the bacterial culture or DNA.

### Application of LAMP assays on a large scale of bacterial isolates

One thousand and eighty-two isolates were subjected to detection by the integron-screening LAMP and standard PCR assays as aforementioned (Table 1). Optimal parameter was used with LAMP reaction processed at 65°C for 45 min. Heating and isothermal amplification were separately performed on the heating block and water bath. Positive LAMP reactions were measured by several qualitative criteria as follows: 1. Observation of white magnesium pyrophosphate precipitates at the bottom of microfuge tubes, which were generated during the strand displacement auto-cycling reaction; 2. Determination by staining with a 1/10 dilution of SYBR Green I and visualization of a positive reaction both colorimetrically by the naked eye as well as imaging under an ultraviolet (UV) light source [51]. In addition, LAMP and PCR amplicons were also evaluated by electrophoresis as mentioned above. These experiments were performed in triplicates to ensure reproducibility.

## References

[1] Xu Z, Li L, Shirtliff M, Peters B, Li B, Peng Y, Alam M, Yamasaki S, Shi L (2011). Resistance class 1 integron in clinical methicillin-resistant *Staphylococcus aureus* strains in Southern China, 2001–2006. Clin Microbiol Infect.

[2] Xu Z, Li L, Zhao X, Chu J, Li B, Shi L, Su J, Shirtliff M (2011). Development and application of a novel multiplex polymerase chain reaction (PCR) assay for rapid detection of various types of staphylococci strains. Afr J Microbiol Res.

[3] Zhong N, Gui Z, Xu L, Huang J, Hu K, Gao Y, Zhang X, Xu Z, Su J, Li B (2013). Solvent-free enzymatic synthesis of 1,3-Diacylglycerols by direct esterification of glycerol with saturated fatty acids. Lip Heal Dis.

[4] Xu Z, Li L, Shi L, Shirtliff M (2011). Class 1 integron in staphylococci. Mol Biol Rep.

[5] Stokes HW, Hall RM (1989). A novel family of potentially mobile DNA elements encoding site-specific gene-integration functions: integrons. Mol Microbiol.

[6] Hall RM, Stokes HW (1993). Integrons: novel DNA elements which capture genes by site-specific recombination. Genetica.

[7] Hall RM, Brown HJ, Brookes DE, Stokes HW (1994). Integrons found in different locations have identical 5b ends but variable 3b ends. J Bacteriol.

[8] Hall RM, Collis CM (1995). Mobile gene cassettes and integrons: capture and spread of genes by site-specific recombination. Mol Microbiol.

[9] Collis CM, Grammaticopoulos G, Briton J, Stokes HW, Hall RM (1993). Site-specific insertion of gene cassettes into integrons. Mol Microbiol.

[10] Collis CM, Hall RM (1995). Expression of antibiotic resistance genes in the integrated cassettes of integrons. Antimicrob Agents Chemother.

[11] Collis CM, Kim MJ, Stokes HW, Hall RM (1995). Binding of the purified integron DNA integrase *Intl1* to integron- and cassette-associated recombination sites. Mol Microbiol.

[12] Hall RM, Collis CM (1998). Antibiotic resistance in gram-negative bacteria: the role of gene cassettes and integrons. Drug Res Updates.

[13] Hall RM, Collis CM, Kim MJ, Partridge SR, Recchia GD, Stokes HW (1999). Mobile gene cassettes and integrons in evolution. Ann N Y Acad Sci.

[14] Stokes HW, O’Gorman DB, Recchia GD, Parsekhian M, Hall RM (1997). Structure and function of 59-base element recombination sites associated with mobile gene cassettes. Mol Microbiol.

[15] Boucher Y, Labbate M, Koenig JE, Stokes HW (2007). Integrons: mobilizable platforms that promote genetic diversity in bacteria. Trends Microbiol.

[16] Fluit AC, Schmitz FJ (1999). Class 1 integrons, gene cassettes, mobility, and epidemiology. Eur J Clin Microbiol Infect Dis.

[17] Fluit AC, Schmitz FJ (2004). Resistance integrons and super-integrons. Clin Microbiol Infect.

[18] Mazel D (2006). Integrons: agents of bacterial evolution. Nat Rev Microbiol.

[19] Labbate M, Case RJ, Stokes HW (2009). The Integron/Gene Cassette System: An Active Player in Bacterial Adaptation.

[20] Francia MV, Zabala JC, de la Cruz F, Garcia Lobo JM (1999). The *IntI1* integron integrase preferentially binds single stranded DNA of the *attC* site. J Bacteriol.

[21] Segal H, Francia MV, Lobo JM, Elisha G (1999). Reconstruction of an active integron recombination site after integration of a gene cassette at a secondary site. Antimicrob Agents Chemother.

[22] Mazel D, Dychinco B, Webb VA, Davies J (2000). Antibiotic resistance in the ECOR collection: integrons and identification of a novel *aad* gene. Antimicrob Agents Chemother.

[23] Recchia GD, Hall RM (1995). Gene cassettes, a new class of mobile element. Microbiol.

[24] Xu Z, Li L, Shirtliff M, Alam M, Yamasaki S, Shi L (2009). Occurrence and characteristics of class 1 and 2 integrons in *Pseudomonas aeruginosa* isolates from patients in Southern China. J Clin Microbiol.

[25] Rowe-Magnus DA, Mazel D (2001). Integrons: natural tools for bacterial genome evolution. Curr Opin Microbiol.

[26] Wang L, Li Y, Xu Z, Zhong Q (2012). Development and application of a simple loop-mediated isothermal amplification method on rapid detection of *Listeria monocytogenes* strains. Mol Biol Rep.

[27] Notomi T, Okayama H, Masubuchi H, Yonekawa T, Watanabe K, Amino N, Hase T (2000). Loop-mediated isothermal amplification of DNA. Nucleic Acids Res.

[28] Mori Y, Nagamine K, Tomita N (2001). Detection of loop-mediated isothermal amplification reaction by turbidity derived from magnesium pyrophosphate formation. Biochem Bioph Res Co.

[29] Nagamine K, Watanabe K, Ohtsuka K (2001). Loop-mediated isothermal amplification reaction using a nondenatured template. Clin Chem.

[30] Zhao X, Li Y, Wang L, You L, Xu Z, Li L, He X, Liu Y, Wang J, Yang L (2010). Development and application of a loop-mediated isothermal amplification method on rapid detection *Escherichia coli* O157 strains from food samples. Mol Biol Rep.

[31] Zhao X, Wang L, Chu J, Li Y, Li Y, Xu Z, Li L, Shirtliff M, He X, Liu Y, Wang J, Yang L (2010). Development and application of a rapid and simple loop-mediated isothermal amplification method for food-borne *Salmonella* detection. Food Sci Biotechnol.

[32] Zhao X, Wang L, Chu J, Li Y, Li Y, Xu Z, Li L, Shirtliff M, He X, Liu Y, Wang J, Yang L (2010). Rapid detection of *Vibrio parahaemolyticus* strains and virulent factors by loop-mediated isothermal amplification assays. Food Sci Biotechnol.

[33] Zhao X, Wang L, Li Y, Xu Z, Li L, He X, Liu Y, Wang J, Yang L (2011). Development and application of a loop-mediated isothermal amplification method on rapid detection of *Pseudomonas aeruginosa* strains. World J Microbiol Biotechnol.

[34] Horisaka T, Fujita K, Iwata T, Nakadai A, Okatani AT, Horikita T, Taniguchi T, Honda E, Yokomizo Y, Hayashidani H (2004). Sensitive and specific detection of *Yersinia pseudotuberculosis* by loop-mediated isothermal amplification. J Clin Microbiol.

[35] Ohtsuka M, Yanagawa K, Takatori K (2005). Detection of *Salmonella enterica* in naturally contaminated liquid eggs by loop-mediated isothermal amplification, and characterization of *Salmonella* isolates. Appl Environ Microbiol.

[36] Okamura M, Ohba Y, Kikuchi S, Suzuki A, Tachizaki H, Takehara K, Ikedo M, Kojima T, Nakamura M (2008). Loop-mediated isothermal amplification for the rapid, sensitive, and specific detection of the O9 group of *Salmonella* in chickens. Vet Microbiol.

[37] Ueda S, Kuwabara Y (2009). The rapid detection of *Salmonella* from food samples by Loop-mediated isothermal amplification (LAMP). Biocontrol Sci.

[38] Chen S, Ge B (2010). Development of a *toxR*-based loop-mediated isothermal amplification assay for detecting *Vibrio parahaemolyticus*. BMC Microbiol.

[39] Nemoto J, Sugawara C, Akahane K, Hashimoto K, Kojima T, Ikedo M, Konuma H, Hara-Kudo Y (2009). Rapid and specific detection of the thermostable direct hemolysin gene in *Vibrio parahaemolyticus* by loop-mediated isothermal amplification. J Food Prot.

[40] Yamazaki W, Ishibashi M, Kawahara R, Inoue K (2008). Development of a loop-mediated isothermal amplification assay for sensitive and rapid detection of *Vibrio parahaemolyticus*. BMC Microbiol.

[41] Yamazaki W, Kumeda Y, Misawa N, Nakaguchi Y, Nishibuchi M (2010). Development of a loop-mediated isothermal amplification assay for sensitive and rapid detection of the *tdh* and *trh* genes of *Vibrio parahaemolyticus* and related *Vibrio* species. Appl Environ Microbiol.

[42] Wang L, Zhao X, Chu J, Li Y, Li Y, Li C, Xu Z, Zhong Q (2011). Application of an improved loop-mediated isothermal amplification detection of *Vibrio parahaemolyticus* from various seafood samples. Afr J Microbiol Res.

[43] Xu Z, Li L, Chu J, Peters B, Harris M, Li B, Shi B, Shirtliff M (2012). Development and application of loop-mediated isothermal amplification assays on rapid detection of various types of staphylococci strains. Food Res Int.

[44] Parida M, Horioke K, Ishida H (2005). Rapid detection and differentiation of dengue virus serotypes by a real-time reverse transcription-loop-mediated isothermal amplification assay. J Clin Microbiol.

[45] Kaneko H, Kawana T, Fukushima E, Suzutani T (2007). Tolerance of loop-mediated isothermal amplification to a culture medium and biological substances. J Biochem Bioph Meth.

[46] You R, Gui Z, Xu Z, Shirtliff M, Yu G, Zhao X, Shi L, Li B, Su J, Li L (2012). Methicillin-Resistance *Staphylococcus aureus* Detection by an improved rapid PCR assay. Afr J Microbiol Res.

[47] Xu Z, Shi L, Zhang C, Zhang L, Li X, Cao Y, Li L, Yamasaki S (2007). Nosocomial infection caused by class 1 integron-carrying *Staphylococcus aureus* in a hospital in South China. Clin Microbiol Infect.

[48] Xu Z, Shi L, Alam M, Li L, Yamasaki S (2008). Integron-bearing methicillin-resistant coagulase-negative staphylococci in South China, 2001–2004. FEMS Microbiol Lett.

[49] Xu Z, Li L, Alam M, Yamasaki S, Shi L (2008). First confirmation of integron-bearing methicillin-resistant *Staphylococcus aureus*. Curr Microbiol.

[50] Xu Z, Li L, Shirtliff M, Peters B, Peng Y, Alam M, Yamasaki S, Shi L (2010). First report of class 2 integron in clinical *Enterococcus faecalis* and class 1 integron in *Enterococcus faecium* in South China. Diag Microbiol Infect Dis.

[51] Xu Z, Gui Z, Zhao X, Zhang Y, He X, Li W, Yang L (2012). Expression and purification of gp41-gp36 fusion protein and application in serological screening assay of HIV-1 and HIV-2. Afr J Microbiol Res.

[52] Deng Y, Liu J, Peters BM, Chen L, Miao J, Li B, Li L, Chen D, Yu G, Xu Z, Shirtliff ME (2014). Antimicrobial resistance investigation on *Staphylococcus* strains in a local hospital in Guangzhou, China, 2001–2010. Microb Drug Resist.

